# Predictors of exclusive breastfeeding cessation among employed mothers in Mogadishu, Somalia: A cross-sectional study

**DOI:** 10.1186/s12884-026-09472-9

**Published:** 2026-06-19

**Authors:** Nasteho Mohamud Mudei, Fartun Abdullahi Hassan Orey, Bashiru Garba, Abdiwali Mohamed Hussein, Abdullahi Abdirahman Omar, Khadra Hassan Mohamud

**Affiliations:** 1 Department of Obstetrics & Gynecology, Dr. Sumait Hospital, SIMAD University, Mogadishu, Somalia; 2 Department of Pediatrics and Child Health, Dr. Sumait Hospital, SIMAD University, Mogadishu, Somalia; 3 SIMAD Institute for Global Health, SIMAD University, Mogadishu, Somalia; 4 Department of Veterinary Public Health and Preventive Medicine, Faculty of Veterinary Medicine, Usmanu Danfodiyo University, Sokoto, Nigeria; 5 Department of Internal Medicine, Dr. Sumait Hospital, SIMAD University, Mogadishu, Somalia; 6 Department of Ophthalmology, Dr. Sumait Hospital, SIMAD University, Mogadishu, Somalia

**Keywords:** Exclusive breastfeeding cessation, Employed mothers, Mogadishu, Somalia, Maternity leave, Workplace support

## Abstract

**Background:**

Exclusive breastfeeding (EBF) is critical for infant health and development during the first six months of life. However, employed mothers often face workplace-related challenges that contribute to EBF cessation before the recommended six months. Evidence specifically examining predictors of EBF cessation among employed mothers in Somalia remains limited. This study aimed to assess the prevalence and predictors of EBF cessation among employed mothers in Mogadishu, Somalia.

**Methods:**

A quantitative, descriptive cross-sectional study was conducted in Mogadishu, Somalia, from August 20, 2024, to October 20, 2024, among 303 employed mothers with children aged 6–24 months. Participants were selected using stratified random sampling across four occupational categories. Data were collected through face-to-face interviews using a structured questionnaire adapted from World Health Organization infant and young child feeding tools and the Somalia Health and Demographic Survey. Bivariable and multivariable logistic regression analyses were performed, and crude odds ratios (CORs) and adjusted odds ratios (AORs) with 95% confidence intervals (CIs) were reported.

**Results:**

The prevalence of EBF cessation was 76.2% (231/303; 95% CI: 70.3%–81.2%). Among mothers with available cessation timing data, the largest proportion stopped EBF in the third month (109/229; 47.6%). In multivariable analysis, flexible working time was associated with lower odds of EBF cessation (AOR = 0.47; 95% CI: 0.23–0.96), and family support was strongly protective (AOR = 0.07; 95% CI: 0.03–0.16). Receiving paid maternity leave showed higher odds of EBF cessation but did not reach statistical significance after adjustment (AOR = 2.52; 95% CI: 0.97–6.54). Lactation breaks showed a non-significant protective trend (AOR = 0.53; 95% CI: 0.24–1.19).

**Conclusion:**

EBF cessation was highly prevalent among employed mothers in Mogadishu. Flexible working time and family support were protective, while paid maternity leave showed a positive but non-significant association with cessation after adjustment, possibly reflecting short leave duration and early return to full-time work. Workplace policies that extend practical maternity protection, improve work flexibility, provide breastfeeding spaces, and strengthen family and employer support are needed.

## Introduction

 Breastfeeding is a fundamental component of the 2030 Agenda for Sustainable Development Goals (SDGs) and is closely linked to several SDG targets, particularly Goal 3, which aims to reduce preventable maternal and neonatal mortality [1]. The World Health Organization (WHO) recommends exclusive breastfeeding (EBF) during the initial six months of an infant’s life, followed by continued breastfeeding in conjunction with appropriate complementary foods for up to two years or more [2]. 

Despite global promotion and clear WHO recommendations, many low- and middle-income countries struggle to achieve sustained EBF rates. National survey data from the Somalia Health and Demographic Survey (SDHS) show that the EBF prevalence in Somalia was 30.7% for infants under six months in 2020 [3]. Somalia has one of the highest neonatal and under-five mortality rates globally, with an estimated 117 deaths per 1,000 live births among children under five [4]. Breastfeeding initiation and exclusivity remain suboptimal in Somalia, with national and regional reports showing persistent gaps in early initiation and EBF practice [3, 5]. 

Early cessation of EBF is influenced by a complex interplay of individual, social, cultural, health-service, and structural factors. Maternal employment, particularly when maternity leave is insufficient or workplace support is lacking, has been identified as a major contributor to EBF cessation [6, 7]. Employed mothers may face barriers that differ from those of non-employed mothers, including fixed work schedules, distance between workplace and infant, short maternity leave, limited breastfeeding spaces, and lack of practical employer support. In Sri Lanka, continuation of EBF up to six months was challenged by maternal workload and structural barriers [8]. 

Across multiple low- and middle-income countries, including Brazil, Ecuador, Ghana, Kenya, and the Somali region of Ethiopia, employment-related constraints, shorter maternity leave, and inflexible work conditions have been associated with premature cessation of EBF [9–13]. For children of employed mothers, inadequate EBF may increase vulnerability to childhood illness, reduce immunological protection, and weaken optimal maternal-infant bonding during a critical developmental period [14]. 

Although EBF has been widely studied globally, evidence from Somalia remains limited, particularly among employed mothers in formal and semi-formal workplace settings. Therefore, this study was undertaken to determine the prevalence and predictors of EBF cessation among employed mothers in Mogadishu, Somalia, and to identify actionable workplace-related factors that may inform maternal and child health policy.

## Methods

### Study design and setting

A quantitative, descriptive, community-based cross-sectional study was conducted in Mogadishu, the capital city of Somalia, from August 20, 2024, to October 20, 2024. A cross-sectional design was chosen because it is appropriate for estimating the prevalence of EBF cessation and identifying associated factors at a single point in time. Mogadishu is the largest urban center of Somalia and is characterized by a mixed economy dominated by trade, services, non-governmental organizations, and government employment. According to the 2019 Somali Labor Force Survey, approximately 242,000 women of reproductive age were employed in government and private sectors [15]. A rapid pre-study survey identified approximately 3,400 eligible employed mothers.

### Study population

The study population consisted of employed mothers working in both public and private sectors in Mogadishu who had children aged 6–24 months. Mothers with children in this age range were included because all children had passed the six-month age threshold required to determine whether EBF was continued for the recommended duration or stopped before six months.

### Inclusion and exclusion criteria

Employed mothers aged 18 years and above who had a child aged 6–24 months, were working either full-time or part-time, and provided written informed consent were included. Mothers who were unable to participate in the interview because of severe illness, cognitive impairment, hearing difficulty, or inability to provide informed consent were excluded.

### Sample size and sampling technique

The required sample size was determined using a single-population proportion formula, assuming a 75% prevalence of EBF cessation, a 95% confidence level, and a 5% margin of error. After accounting for a 5% non-response rate, the final sample size was 303.

Study participants were selected using stratified random sampling across four occupational categories: health workers, government/public officers, academic workers, and business workers. Based on the final dataset, the sample included 84 health workers, 77 government/public officers, 64 academic workers, and 78 business workers. Simple random sampling was applied within each stratum. The earlier Fig. 1 callout was removed because no figure is included in the final submission.

### Study variables

The dependent variable was EBF cessation, coded as 1 = discontinued EBF before six months and 0 = continued EBF to six months. EBF cessation was defined as the introduction of any food or liquid other than breast milk before the infant reached six months of age, except oral rehydration solution, vitamins, minerals, or prescribed medicines.

Independent variables included sociodemographic characteristics, work-related factors, obstetric variables, and health-service-related variables. Sociodemographic variables included age, educational level, marital status, number of children, and household monthly income. Work-related variables included type of organization, working hours per week, duty shift, flexible working time, paid maternity leave, maternity leave duration, availability of a breastfeeding room, lactation breaks, lactation break time, daycare near the workplace, and distance between child and workplace. Obstetric and health-service variables included antenatal care attendance, breastfeeding counseling during ANC, time of breastfeeding initiation, mode of delivery, postnatal care service, family support, and reasons for stopping EBF.

Receipt of paid maternity leave was measured as a binary variable (yes/no), while maternity leave duration was analyzed descriptively to contextualize the finding for paid leave. Duration was not retained as a separate predictor in the final multivariable model because it was applicable mainly to mothers who received paid leave and some duration categories contained small numbers.

### Data collection process and measurements

Data were collected using a structured questionnaire adapted from WHO infant and young child feeding tools and the Somalia Health and Demographic Survey [3, 16]. Additional items were developed from related literature to capture context-specific work-related factors among employed mothers in Mogadishu.

The questionnaire was initially prepared in English and translated into Somali by a bilingual health professional. The Somali version was reviewed by the research team to ensure conceptual equivalence, cl arity, and cultural appropriateness. Data were collected through face-to-face interviews by trained data collectors with health-science backgrounds under supervision of the research team. Each interview lasted approximately 20–25 min.

Content validity was supported through expert review, and the questionnaire was pretested among 15 employed mothers outside the main study area. Based on pretesting feedback, unclear wording was simplified and response categories related to breastfeeding initiation and workplace breastfeeding support were clarified. To improve recall accuracy, interviewers used a standardized EBF definition and asked month-specific questions about the timing of introduction of foods or liquids other than breast milk.

### Data analysis

Data were checked for completeness, coded, and entered into SPSS version 20 for analysis. Descriptive statistics were used to summarize the data, and results are presented using frequencies and percentages. Bivariable and multivariable binary logistic regression analyses were conducted to identify factors associated with EBF cessation. Variables with *p* ≤ 0.25 in bivariable analysis and variables considered relevant based on prior evidence were considered for multivariable logistic regression. Associations were expressed as crude odds ratios (CORs), adjusted odds ratios (AORs), and 95% confidence intervals (CIs). Statistical significance was set at *p* < 0.05.

### Ethical approval

Ethical approval was obtained from the Center of Research and Development of SIMAD University (EC000146). All participants provided written informed consent after receiving an explanation of voluntary participation, their right to withdraw, and confidentiality assurances.

## Results

### Prevalence of exclusive breastfeeding cessation

More than three-fourths of employed mothers had discontinued EBF before six months (76.2%; 231/303; 95% CI: 70.3%–81.2%). Among mothers with available cessation timing data, the largest proportion stopped EBF in the third month (109/229; 47.6%) (Table 4).

### Sociodemographic and socioeconomic characteristics

A total of 303 mothers were included in the study. Most participants were aged 26–35 years (60.7%), 57.8% had completed a bachelor’s degree, 72.9% were married, and 35.6% had three children. The largest proportion of households reported a monthly income of $300–499 (44.2%) (Table 1).

**Table 1:** Sociodemographic and socio-economic characteristics of respondents

Variable	Category	Number	Percentage (%)
Age	18–25	60	19.8%
	26–35	184	60.7%
	36–45	59	19.5%
Educational Level	Primary	20	6.6%
	Secondary	68	22.4%
	Bachelor	175	57.8%
	Master and above	40	13.2%
Marital Status	Married	221	72.9%
	Divorced	72	23.8%
	Widowed	10	3.3%
Number of Children	One	56	18.5%
	Two	92	30.4%
	Three	108	35.6%
	More than 4	47	15.5%
Household Monthly Income	$100–299	44	14.5%
	$300–499	134	44.2%
	$500–999	94	31.0%
	>$1000	31	10.2%

### Work-related factors

The highest rates of EBF cessation were observed among business workers (85.9%) and academic workers (79.7%). Full-time workers had higher cessation than part-time workers (78.1% vs. 58.6%). Mothers without flexible working time had higher cessation than those with flexible working time (87.1% vs. 54.5%). EBF cessation was also higher among mothers without a breastfeeding room (80.6%) than among those with a breastfeeding room (25.0%). Mothers who received paid maternity leave had higher cessation than those who did not (78.0% vs. 62.9%), while longer leave duration showed descriptively lower cessation at six months (57.1%) (Table 2).

**Table 2:** Work-related factors and EBF cessation among employed mothers in Mogadishu, Somalia

Variable	Category	Total (*n*)	Discontinued *n* (%)	Continued *n* (%)
Type of organization	Health worker	84	55 (65.5)	29 (34.5)
	Government/public officer	77	58 (75.3)	19 (24.7)
	Academic worker	64	51 (79.7)	13 (20.3)
	Business	78	67 (85.9)	11 (14.1)
Working hours/week	Full-time (> 35 h/week)	274	214 (78.1)	60 (21.9)
	Part-time (< 35 h/week)	29	17 (58.6)	12 (41.4)
Duty shift	Day	266	201 (75.6)	65 (24.4)
	Night	17	15 (88.2)	2 (11.8)
	Mixed	20	15 (75.0)	5 (25.0)
Flexible working time	Yes	101	55 (54.5)	46 (45.5)
	No	202	176 (87.1)	26 (12.9)
Paid maternity leave	Yes	268	209 (78.0)	59 (22.0)
	No	35	22 (62.9)	13 (37.1)
Maternity leave duration*	1 month	23	14 (60.9)	9 (39.1)
	2 months	42	33 (78.6)	9 (21.4)
	3 months	129	105 (81.4)	24 (18.6)
	4 months	100	73 (73.0)	27 (27.0)
	6 months	7	4 (57.1)	3 (42.9)
Breastfeeding room available	Yes	24	6 (25.0)	18 (75.0)
	No	279	225 (80.6)	54 (19.4)
Lactation breaks at work	Yes	212	160 (75.5)	52 (24.5)
	No	91	71 (78.0)	20 (22.0)
Lactation break time	Not fixed	73	59 (80.8)	14 (19.2)
	30 min	39	21 (53.8)	18 (46.2)
	1 h	123	105 (85.4)	18 (14.6)
	2 h	68	46 (67.6)	22 (32.4)
Daycare near workplace	Yes	4	1 (25.0)	3 (75.0)
	No	299	230 (76.9)	69 (23.1)
Distance child-workplace	10–20 min	65	33 (50.8)	32 (49.2)
	20–30 min	134	114 (85.1)	20 (14.9)
	> 30 min	104	84 (80.8)	20 (19.2)

﻿*Maternity leave duration was summarized descriptively and was not retained as a separate predictor in the final multivariable model

### Obstetric and health-service-related factors

EBF cessation varied by ANC attendance, breastfeeding counseling during ANC, time of breastfeeding initiation, mode of delivery, and postnatal care service. Returning to work was the most frequently reported reason for stopping EBF (170 responses), followed by perceived insufficient milk (85 responses). Reasons for stopping were recorded as multiple responses (Tables 3 and 4).

**Table 3:** Obstetric and health-service-related factors and EBF cessation among employed mothers in Mogadishu, Somalia

Variable	Category	Total (*n*)	Discontinued *n* (%)	Continued *n* (%)
Number of ANC visits	None	1	1 (100.0)	0 (0.0)
	1–2 visits	83	70 (84.3)	13 (15.7)
	>=3 visits	219	160 (73.1)	59 (26.9)
Breastfeeding counseling during ANC	Yes	233	170 (73.0)	63 (27.0)
	No	70	61 (87.1)	9 (12.9)
Time of breastfeeding initiation	Within first hour	70	34 (48.6)	36 (51.4)
	First 2 h	16	12 (75.0)	4 (25.0)
	1–3 h	133	113 (85.0)	20 (15.0)
	After 4 h	84	72 (85.7)	12 (14.3)
Mode of delivery	Vaginal	227	177 (78.0)	50 (22.0)
	Cesarean section	76	54 (71.1)	22 (28.9)
Postnatal care service	Yes	147	97 (66.0)	50 (34.0)
	No	156	134 (85.9)	22 (14.1)

**Table 4:** Timing and main reported reason for EBF cessation among mothers who discontinued EBF

Variable/category	*n*	%
Timing among mothers with known cessation timing (*n* = 229)		
1st month	24	10.5
2nd month	44	19.2
3rd month	109	47.6
4th month	40	17.5
5th month	9	3.9
6th month	3	1.3
Main reported reason for stopping EBF among discontinued mothers (*n* = 231)		
Returning to work	152	65.8
Perceived insufficient milk	42	18.2
Family pressure to give water or other liquids	16	6.9
Time shortage	12	5.2
Nipple pain	9	3.9

EBF cessation timing was calculated only among mothers who discontinued EBF and had available cessation timing data. Main reason for stopping EBF was calculated only among mothers who discontinued EBF before six months.

### Factors associated with EBF cessation

In the multivariable logistic regression model, flexible working time was significantly associated with lower odds of EBF cessation (AOR = 0.47; 95% CI: 0.23–0.96). Family support was also strongly protective (AOR = 0.07; 95% CI: 0.03–0.16). Receiving paid maternity leave showed higher odds of EBF cessation but did not reach statistical significance after adjustment (AOR = 2.52; 95% CI: 0.97–6.54). Lactation breaks showed a non-significant protective trend (AOR = 0.53; 95% CI: 0.24–1.19) (Table 5).

**Table 5:** Logistic regression analysis of factors associated with EBF cessation among employed mothers in Mogadishu, Somalia

Variable	Category	COR (95% CI)	AOR (95% CI)
Educational status	Primary	1.00 (ref)	1.00 (ref)
	Secondary	0.91 (0.23–3.66)	1.35 (0.23–7.95)
	Bachelor	0.51 (0.14–1.82)	1.13 (0.19–6.51)
	Master and above	0.37 (0.09–1.48)	1.40 (0.20-10.01)
Type of organization	Government/public officer	1.00 (ref)	1.00 (ref)
	Academic worker	1.29 (0.58–2.86)	0.61 (0.21–1.75)
	Business	2.00 (0.88–4.54)	2.11 (0.65–6.88)
	Health worker	0.62 (0.31–1.23)	0.71 (0.28–1.78)
Flexible working time	No	1.00 (ref)	1.00 (ref)
	Yes	0.18 (0.10–0.31)	0.47 (0.23–0.96)*
Lactation breaks at work	No	1.00 (ref)	1.00 (ref)
	Yes	0.87 (0.48–1.56)	0.53 (0.24–1.19)
Paid maternity leave	No	1.00 (ref)	1.00 (ref)
	Yes	2.09 (0.99–4.41)	2.52 (0.97–6.54)
Family support	No	1.00 (ref)	1.00 (ref)
	Yes	0.06 (0.03–0.12)	0.07 (0.03–0.16)*

*COR* Crude odds ratio, *AOR* Adjusted odds ratio, *CI* Confidence interval, *ref* reference category

Outcome coded as 1 = EBF cessation before six months and 0 = EBF continuation to six months

* p < 0.05

## Discussion

This study assessed the prevalence and predictors of EBF cessation among employed mothers in Mogadishu, Somalia. The findings showed that EBF cessation was high, with more than three-quarters of employed mothers discontinuing EBF before six months. This finding highlights an important maternal and child health challenge in an urban Somali context where return to work and workplace conditions may affect mothers’ ability to continue EBF for the recommended duration. Similar findings have been documented in studies from Bangladesh, Ghana, and Ethiopia, where maternal employment and related barriers were associated with suboptimal EBF continuation [6, 17, 18]. 

Workplace factors were important in explaining EBF cessation. Flexible working time was significantly associated with lower odds of EBF cessation, suggesting that flexibility may allow mothers to breastfeed directly, express breast milk, or remain closer to their infants. In contrast, full-time work, limited workplace breastfeeding spaces, and lack of practical support were associated with higher descriptive levels of EBF cessation. Evidence from workplace breastfeeding support studies also shows that lactation spaces, workplace flexibility, and employer-based support can improve breastfeeding outcomes [19]. In Mogadishu, many women work in informal or poorly protected employment settings, which may limit access to formal breastfeeding support [20]. 

An unexpected positive association between paid maternity leave and EBF cessation was observed, although the association did not reach statistical significance after adjustment. This finding should not be interpreted as evidence that paid maternity leave is harmful. Rather, it may reflect the local employment context in Mogadishu, where paid maternity leave is often short and followed by return to full-time work before completion of the recommended six months of EBF. Mothers without formal paid leave may include self-employed or informally employed women with greater breastfeeding flexibility. Therefore, the finding likely reflects the insufficiency and practical implementation of maternity leave rather than a negative effect of maternity leave itself. This interpretation is consistent with broader evidence showing that longer paid maternity leave can improve breastfeeding outcomes [21, 22]. 

Although lactation breaks showed a protective direction, the association was not statistically significant after adjustment. This finding should therefore be interpreted cautiously and should not be presented as a proven independent predictor. The limited effect of lactation breaks may reflect the fact that breaks alone are insufficient if they are not accompanied by breastfeeding rooms, milk-expression facilities, supportive supervisors, or flexible work schedules.

Family support was strongly protective against EBF cessation. This is important in the Somali context, where partners and extended family members often play a major role in childcare and household support. Mothers who received encouragement and help from partners or extended family members were less likely to stop breastfeeding early. This is consistent with evidence from Indonesia, where family and husband support were strongly associated with continued breastfeeding among employed mothers [23]. Interventions to improve EBF continuation among employed mothers should therefore include family members, employers, health workers, and community structures.

These findings have practical implications for maternal and child health policy. Supporting EBF among employed mothers requires more than health education. It requires workplace policies that combine adequate maternity leave duration, flexible working arrangements, private breastfeeding or milk-expression spaces, supportive supervisors, and family-centered counseling through antenatal and postnatal care services.

### Study limitations

This study has several limitations. The cross-sectional design restricts causal inference and prevents examination of changes in breastfeeding practices over time. Breastfeeding practices were self-reported and may have been affected by recall bias, particularly among mothers recalling feeding practices from earlier infancy. To reduce recall error, interviewers used a standardized EBF definition and asked month-specific questions about the timing of introduction of foods or liquids other than breast milk. Social desirability bias may also have influenced reporting of EBF practices. Some variables, including workplace breastfeeding policies, quality of lactation breaks, and exact enforcement of maternity leave, were not measured objectively. Finally, the findings may not be generalizable to unemployed mothers or to mothers outside Mogadishu.

## Conclusion

EBF cessation among employed mothers in Mogadishu was high. Flexible working time and family support were associated with lower odds of EBF cessation. Receiving paid maternity leave showed higher but non-significant adjusted odds of cessation, likely reflecting short leave duration and early return to full-time work rather than a harmful effect of maternity leave itself. Although lactation breaks showed a non-significant protective trend, the findings highlight the need for breastfeeding-friendly workplace policies that combine adequate maternity leave duration, flexible work arrangements, private breastfeeding spaces, and practical family and employer support.

## Data Availability

The data supporting the findings of this study are available from the corresponding author upon reasonable request.

## Abbreviations

ANC: Antenatal care
AOR: Adjusted odds ratio
CI: Confidence interval
COR: Crude odds ratio
CS: Cesarean section
EBF: Exclusive breastfeeding
OR: Odds ratio
PNC: Postnatal care
SDG: Sustainable Development Goal
SDHS: Somalia Health and Demographic Survey
SPSS: Statistical Package for the Social Sciences
WHO: World Health Organization
